# ﻿Two new species of the genus *Viscosia* de Man, 1890 (Nematoda, Enoplea, Enoplida, Oncholaimidae) from the intertidal zone of the Yellow Sea, China

**DOI:** 10.3897/zookeys.1231.142078

**Published:** 2025-03-11

**Authors:** Lingyun Sun, Huimin Gu, Yong Huang

**Affiliations:** 1 College of Agriculture and Biology, Liaocheng University, Liaocheng 252059, China Liaocheng University Liaocheng China

**Keywords:** Biodiversity, free-living marine nematodes, Huangdao coast, Qingdao, Rizhao coast, Shandong Province, taxonomy

## Abstract

Two new species of *Viscosia* from the intertidal zone along the Yellow Sea are described and illustrated. *Viscosiamedia***sp. nov.** is characterized by a heavily cuticularized and relatively shallow buccal cavity with stubby teeth; cephalic setae 7–8 µm long; amphidial fovea invisible; slender spicules almost straight, cephalated proximally and conical distally; and tail conical, straight in males and slightly bent ventrally in females. *Viscosiasinica***sp. nov.** is characterized by a relatively large amphidial fovea, conico-cylindrical tail with swollen horseshoe-shaped tip, spicules slightly curved ventrally, and 10–12 setae surrounding the cloaca, each 3–4 µm long. The basic morphological data of males of 26 valid species in *Viscosia* with a body length of 1–2.9 mm are presented.

## ﻿Introduction

Free-living marine nematodes are the most widespread and abundant meiofaunal group in marine benthic habitats (Heip et al. 1982). They play a significant role in marine environment and are widely used as pollution indicators in biological monitoring (Lambshead 2004). However, many species of nematodes are still unknown. At present, less than 600 species of free-living marine nematodes have been identified in the Chinese sea area, which is estimated to be less than half of the total species (Sun et al. 2024).

The Yellow Sea is located on the edge of the western Pacific Ocean, between China and the Korean Peninsula. It is a semi-enclosed inland shallow sea basin. Biodiversity surveys and taxonomical studies on nematodes in the Yellow Sea have been carried out in recent years (Huang and Sui 2024; Huang and Zhai 2024; Li et al. 2024). More than 390 species of nematodes have been identified, of which 117 species were new to science (Sun et al. 2024). The new species accounted for 30% of the total known species. However, the total number of nematodes in this sea area is unknown, and new species are routinely found. It is, therefore, important to continue investigating the taxonomy of nematodes in the region. Here, the present paper describes two new species of the genus *Viscosia* de Man, 1890 that were discovered in the intertidal zone of Qingdao and Rizhao, Yellow Sea.

*Viscosia* is a large genus with many species. It occurs in marine, brackish, and freshwater sediments (Smol et al. 2014; Leduc and Zhao 2023). The genus was established by de Man in 1890 as a subgenus of *Oncholaimus* Dujardin, 1845. Later, it was raised to genus by Filipjev in 1918. Wieser and Hopper (1967), Gerlach and Riemann (1974), Filipjev (1918), Smol and Sharma (1984), Smol et al. (2014), and Leduc and Zhao (2023) successively reviewed the genus. Wieser and Hopper (1967) stressed the importance of certain characteristics in species identification, such as the size of amphids, the shape of the buccal cavity and teeth, the arrangement of the male circumanal organs like papillae and bursa, and the shape of the tail. Gagarin (2020) provided the basic morphological features of males of 24 valid species with body length of 1–2 mm within the genus *Viscosia*. Currently, the World Database of Nematodes (Nemys eds. 2024) makes a list of 92 valid species including four freshwater species (*Viscosianicaraguensis* Gerlach, 1957, *V.orientalis* Gagarin, 2020, *V.timmi* Gagarin & Nguyen Thi Thu, 2008 and *V.uipii* Coomans, Vincx & Decraemer, 1985). But in fact, many species were described only based on juveniles or females, and lack the characters of males. In the Chinese sea area, only seven species within *Viscosia* were recorded. They are *Viscosiabandaensis* Kreis, 1932, *V.elegans* (Kreis, 1924) Lorenzen, 1981, *V.franzii* Boucher, 1977, *V.glabra* (Bastian, 1865) de Man, 1890, *V.heterolaima* Smol & Sharma, 1984, *V.longicaudatoides* Nguyen Vu Thanh & Gagarin, 2013 and *V.nuda* Kreis, 1932.

## ﻿Material and methods

The meiofauna samples were collected from the top sediment layer (0–8 cm deep) using a 2.9 cm diameter sawn-off syringe. The samples were fixed with an equal amount of 10% formalin solution. In the laboratory, samples were stained with 0.1% Rose Bengal for 24 hours. The stained samples were poured through two sieves (500 and 42 µm mesh sizes), and washed with tap water to remove silt and separate macrofauna from meiofauna. The intercepted material in the 42 µm mesh was centrifuged in Ludox-TM (50% colloidal silica, suspension in water; product of Sigma Aldrich Co., USA) with a specific gravity of 1.15 g/ml (de Jonge and Bouwman 1977) to separate meiofauna from the heavier sediment particles. The supernatant obtained after three rounds of centrifugation was poured into the 42 µm mesh to filter Ludox-TM. The sample in the 42 µm mesh was washed into a Petri dish with distilled water and meiofauna was sorted out under a stereoscopic microscope.

Nematodes were transferred into a cavity block containing a solution of 5% glycerol, 5% pure ethanol, and 90% distilled water in volume (McIntyre and Warwick 1984). After ethanol was slowly evaporated, the specimens were mounted in glycerin on permanent slides. The descriptions were made using a differential interference contrast microscope (Leica DM 2500). The photos were taken with a Leica DMC 5400 digital camera. Line drawings were made with the aid of a camera lucida. All measurements were taken using Leica software of LAS X version 3.3.3, and all curved structures were measured along the curved median line. All measurements are in μm. Type specimens were deposited in the Marine Biological Museum of the Chinese Academy of Sciences, Qingdao.

## ﻿Results and discussion

### ﻿Taxonomy

**Class Enoplea Inglis, 1983**

**Order Enoplida Filipjev, 1929**

**Family Oncholaimidae Filipjev, 1916**

#### ﻿Genus Viscosia

Taxon classificationAnimaliaEnoplidaOncholaimidae

de Man, 1890

84C8587D-BAA1-5143-B230-C5DCA3707976

##### Diagnosis

**(according to Smol et al. 2014).** Buccal cavity large, barrel-shaped with three unequal immovable teeth, of which the right subventral tooth largest. Females didelphic-amphidelphic with reflexed ovaries. Spicules short, straight or slightly curved. Gubernaculum absent. Demanian system present and simple, consisting of prolongation of the ovary at the reflexed point that connects it with the intestine through the osmosium.

###### ﻿List of valid species (64 species)

*V.abyssorum* (Allgén, 1933) Wieser, 1953.

syn. *Oncholaimusabyssorum* Allgén, 1933.

*V.angustata* (Cobb, 1890) Kreis, 1934.

syn. *Oncholaimusangustatus* Cobb, 1890.

*V.antarctica* Allgén, 1959.

*V.bandaensilis* Lorenzen, 1981.

syn. *Mononcholaimusbandaensis* Kreis, 1932.

*V.bandaensis* Kreis, 1932.

syn. *Mononcholaimusbandaensis* Kreis, 1932.

*V.bayensis* Keppner, 1987.

*V.brachylaima* Filipjev, 1927.

*V.brevicaudata* Mawson, 1958.

*V.brevilaima* Allgén, 1959.

*V.cobbi* Filipjev, 1918.

*V.coomansi* Smol & Sharma, 1984.

*V.dossena* Leduc & Zhao, 2023.

*V.elegans* (Kreis, 1924) Lorenzen, 1981.

syn. *Mononcholaimuselegans* Kreis, 1924.

*V.elongata* Filipjev, 1922.

*V.epapillosa* Platonova, 1971.

*V.erasmi* Furstenberg & Vincx, 1989.

*V.falklandiae* Allgén, 1959.

*V.filiformis* (Kreis, 1932) Lorenzen, 1981.

syn. *Mononcholaimusfiliformis* Kreis, 1932.

*V.floridana* Keppner, 1987.

*V.franzii* Boucher, 1977.

*V.glabra* (Bastian, 1865) de Man, 1890.

syn. *Oncholaimusglaber* Bastian, 1865.

syn. *Viscosiamicoletzkyi* Chitwood, 1951.

*V.grahami* Allgén, 1959.

*V.hanstroemi* Wieser, 1953.

*V.heterolaima* Smol & Sharma, 1984.

*V.keiensilis* Lorenzen, 1981.

syn. *Mononcholaimuskeiensis* Kreis, 1932.

*V.keiensis* Kreis, 1932.

syn. *Mononcholaimuskeiensis* Kreis, 1932.

*V.langrunensis* (de Man, 1890) Filipjev, 1918.

syn. *Oncholaimuslangrunensis* de Man, 1890.

*V.longicaudatoides* Nguyen Vu Thanh & Gagarin, 2013.

*V.macramphida* Chitwood, 1951.

*V.macrobursata* Keppner, 1987.

*V.megalaima* (Ditlevsen, 1928) Hope & Murphy, 1972.

syn. *Steineriamegalaima* Ditlevsen, 1928.

*V.meridionalis* Kreis, 1932.

*V.microseta* Wieser, 1953.

*V.nicaraguensis* Gerlach, 1957.

syn. *Viscosiapapillatanicaraguensis* Gerlach, 1957.

*V.nona* Filipjev, 1946.

*V.norvegica* (Allgén, 1946) Lorenzen, 1981.

syn. *Mononcholaimusnorvegicus* Allgén, 1946.

*V.nuda* Kreis, 1932.

*V.oncholaimelloides* Wieser & Hopper, 1967.

*V.orientalis* Gagarin, 2020.

*V.papillata* Chitwood, 1951.

*V.papillatoides* Chitwood, 1960.

*V.papillatula* Lorenzen, 1981.

syn. *Mononcholaimuspapillatus* Kreis, 1932.

*V.parafalklandiae* Allgén, 1959.

*V.parapellucida* (Allgén, 1959) Gerlach & Riemann, 1974.

syn. *Viscosiapellucida* Allgén, 1959.

*V.parasetosa* (Kreis, 1932) Lorenzen, 1981.

syn. *Mononcholaimusparasetosus* Kreis, 1932.

*V.pedroensis* Allgén, 1947.

*V.profunda* (Vitiello, 1970) Lorenzen, 1981.

syn. *Mononcholaimusprofundus* Vitiello, 1970.

*V.propinqua* Allgén, 1959.

*V.pseudoglabra* Kreis, 1932.

*V.pygmaea* Nguyen Vu Thanh & Gagarin, 2013.

*V.rectangulata* Wieser, 1953.

*V.rustica* (Kreis, 1929) Lorenzen, 1974.

syn. *Mononcholaimusrusticus* Kreis, 1929.

*V.sedata* Gagarin & Nguyen Vu Thanh, 2007.

*V.separabilis* (Wieser, 1953) Lorenzen, 1981.

syn. *Mononcholaimusseparabilis* Wieser, 1953.

*V.setosa* (Kreis, 1932) Lorenzen, 1981.

syn. *Mononcholaimussetosus* Kreis, 1932.

*V.similis* Allgén, 1959.

*V.stenolaima* Filipjev, 1927.

*V.tenuissima* Allgén, 1959.

*V.timmi* Gagarin & Nguyen Thi Thu, 2008.

*V.tumidula* Wieser, 1959.

*V.uipii* Coomans, Vincx & Decraemer, 1985.

*V.viscosa* (Bastian, 1865) de Man, 1890.

syn. *Mononcholaimusviscosus* Allgén, 1930.

syn. *Oncholaimusviscosus* Bastian, 1865.

*V.viscosula* Lorenzen, 1981.

syn. *Mononcholaimusviscosus* Allgén, 1930.

*V.wieseri* Mawson, 1958.

###### ﻿Species *inquirendae* (38 species)

*V.brachydonta* Allgén, 1959.

*V.brachylaimoides* Chitwood, 1937.

*V.brevidentata* (Vitiello, 1967) Lorenzen, 1981.

syn. *Mononcholaimusbrevidentatus* Vitiello, 1967.

*V.conicaudata* (Kreis, 1932) Lorenzen, 1981.

syn. *Mononcholaimusconicaudatus* Kreis, 1932.

*V.crassa* Kreis, 1932.

*V.cryptodentata* Allgén, 1959.

*V.diodon* (Wieser, 1951) Lorenzen, 1981.

syn. *Mononcholaimusdiodon* (Wieser, 1951) Wieser, 1953.

syn. *Oncholaimellusdiodon* Wieser, 1951.

*V.dubiosa* Kreis, 1932.

*V.fatigans* Filipjev, 1946.

*V.filipjevi* Paramonov, 1929.

*V.gabriolae* (Allgén, 1951) Lorenzen, 1981.

syn. *Mononcholaimusgabriolae* Allgén, 1951.

*V.glaberoides* (Allgén, 1932) Lorenzen, 1981.

syn. *Mononcholaimusglaberoides* Allgén, 1932.

*V.isotonchula* Kreis, 1932.

*V.klatti* (Allgén, 1941) Lorenzen, 1981.

syn. *Mononcholaimusklatti* Allgén, 1941.

*V.leptolaima* Kreis, 1932.

*V.linstowi* (de Man, 1904) Filipjev, 1918.

syn. *Oncholaimuslinstowi* de Man, 1904.

*V.longicaudata* (Kreis, 1932) Lorenzen, 1981.

syn. *Meroviscosialongicaudata* Kreis, 1932.

*V.longidentata* (Schuurmans Stekhoven, 1931) Lorenzen, 1981.

syn. *Mononcholaimuslongidentatus* (Schuurmans Stekhoven & Adam in Schuurmans Stekhoven, 1931) Kreis, 1934.

syn. *Oncholaimuslongidentatus* Schuurmans Stekhoven & Adam in Schuurmans Stekhoven, 1931.

*V.longissima* Filipjev, 1946.

*V.macrorhopalocerca* Kreis, 1932.

*V.minor* Filipjev, 1918.

*V.minudonta* Vitiello, 1970.

*V.nijhoffi* Allgén, 1935.

*V.nordgaardi* Allgén, 1940.

*V.palmae* Schuurmans Stekhoven, 1942.

*V.papillosa* (Eberth, 1863) Kreis, 1934.

syn. *Oncholaimuspapillosus* Eberth, 1863.

*V.paralinstowi* Chitwood, 1937.

*V.parapedroensis* Allgén, 1947.

*V.paridentata* Kreis, 1932.

*V.parva* Kreis, 1929.

*V.pellucida* (Cobb, 1898) Filipjev, 1918.

syn. *Oncholaimuspellucidus* Cobb, 1898.

*V.poseidonica* Belogurov & Belogurova, 1977.

*V.pseudosegmentata* Allgén, 1947.

*V.stenostoma* Platonova, 1971.

*V.strandi* Allgén, 1935.

*V.subantarctica* Allgén, 1959.

*V.tasmaniensis* (Allgén, 1927) Lorenzen, 1981.

syn. *Mononcholaimustasmaniensis* Allgén, 1927.

*V.tenuilaima* Allgén, 1959.

###### ﻿Invalid species

*V.aegyptica* (Steiner, 1921) Schuurmans Stekhoven 1943 = *Oncholaimusaegypticus* Steiner, 1921.

*V.carnleyensis* (Ditlevsen, 1921) Kreis, 1932 = *Viscosiaglabra* (Bastian, 1865) de Man, 1890.

*V.carnleyensistropica* Kreis, 1932 = *V.carnleyensis* (Ditlevsen, 1921) Kreis, 1932.

*V.donsi* Allgén, 1947 = *Oncholaimusdonsi* (Allgén, 1947) Wieser, 1953.

*V.micoletzkyi* Chitwood, 1951 = *Viscosiaglabra* (Bastian, 1865) de Man, 1890.

*V.pacifica* Allgén, 1951 = *Oncholaimusrapax* Kreis, 1932.

*V.papillatanicaraguensis* Gerlach, 1957 = *Viscosianicaraguensis* Gerlach, 1957.

*V.paralangrunensis* Allgén, 1947 = *Oncholaimusparalangrunensis* (Allgén, 1947) Allgén, 1959.

*V.pellucida* Allgén, 1959 = *V.parapellucida* (Allgén, 1959) Gerlach & Riemann, 1974.

*V.scanica* Allgén, 1935 = *Oncholaimusscanicus* (Allgén, 1935) Wieser, 1953.

*V.taboguillensis* Allgén, 1947 = *Adoncholaimustaboguillensis* (Allgén, 1947) Wieser, 1953.

**Remarks.** According to the relevant literature, there are 27 species described only from juveniles or females (five species, *V.brachydonta*, *V.conicaudata*, *V.leptolaima*, *V.macrorhopalocerca* and *V.palmae*, were described only from juveniles; 18 species, *V.brevidentata*, *V.cryptodentata*, *V.fatigans*, *V.filipjevi*, *V.isotonchula*, *V.linstowi*, *V.longidentata*, *V.minor*, *V.papillosa*, *V.paralinstowi*, *V.paridentata*, *V.parva*, *V.pellucida*, *V.poseidonica*, *V.stenostoma*, *V.subantarctica*, *V.tasmaniensis* and *V.tenuilaima*, were described only from females; four species, *V.crassa*, *V.diodon*, *V.longissima* and *V.minudonta*, were described from females and juveniles). These species should be considered as *inquirendae*. According to the original description of *V.brachylaimoides* by Chitwood (1937) and the opinion of Leduc and Zhao (2023), this species has a large left ventrosublateral tooth instead of large right ventrosublateral tooth, indicating that the species does not belong to *Viscosia*. Here, *V.brachylaimoides* is treated as species *inquirendum*. Based on the observation and analysis by Smol and Sharma (1984), the largest ventrosublateral tooth of *V.franzii* is situated on the right side instead of the left as described by Boucher (1977). The species is consistent with the diagnosis of *Viscosia*, and it is a valid species of the genus. Meanwhile, Smol and Sharma considered *V.carnleyensis* as a synonym of *V.glabra*.

#### ﻿ Viscosia media sp. nov.

Taxon classificationAnimaliaEnoplidaOncholaimidae

E291695C-7BC8-5A0D-9E98-074119BC2183

https://zoobank.org/EE875370-C9F8-4025-A4D0-519431DC0893

**Figure 1.**
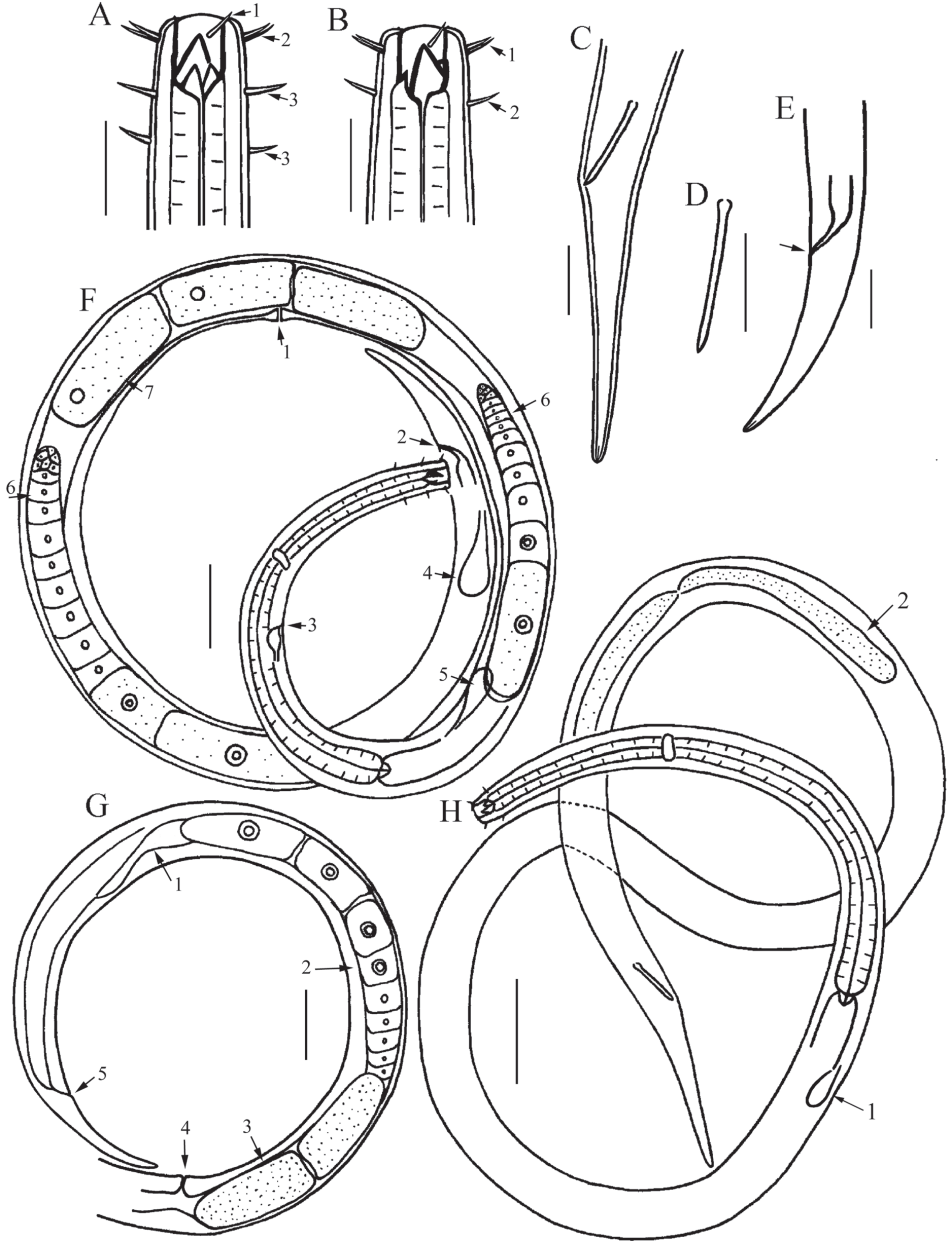
Drawings of *Viscosiamedia* sp. nov. **A** anterior end of holotype, showing outer labial seta (arrow 1), outer labial and cephalic setae (arrow 2), buccal cavity with three teeth, and cervical setae (arrow 3) **B** anterior end of female, showing large right subventral tooth, small dorsal tooth, outer labial and cephalic setae (arrow 1) and cervical setae (arrow 2) **C** posterior end of holotype, showing spicule and tail **D** spicule **E** posterior end of female, showing anus (arrow) and tail **F** entire view of female (arrow 1, vulva; 2, anus; 3, excretory pore; 4, caudal gland; 5, ventral gland; 6, ovary) **G** posterior part of female (arrow 1, Demanian system; 2, ovary; 3, egg; 4, vulva; 5, anus) **H** entire view of male (arrow 1, ventral gland; arrow 2, testis). Scale bars: 20 µm (**A–E**); 50 µm (**F–H**).

**Figure 2.**
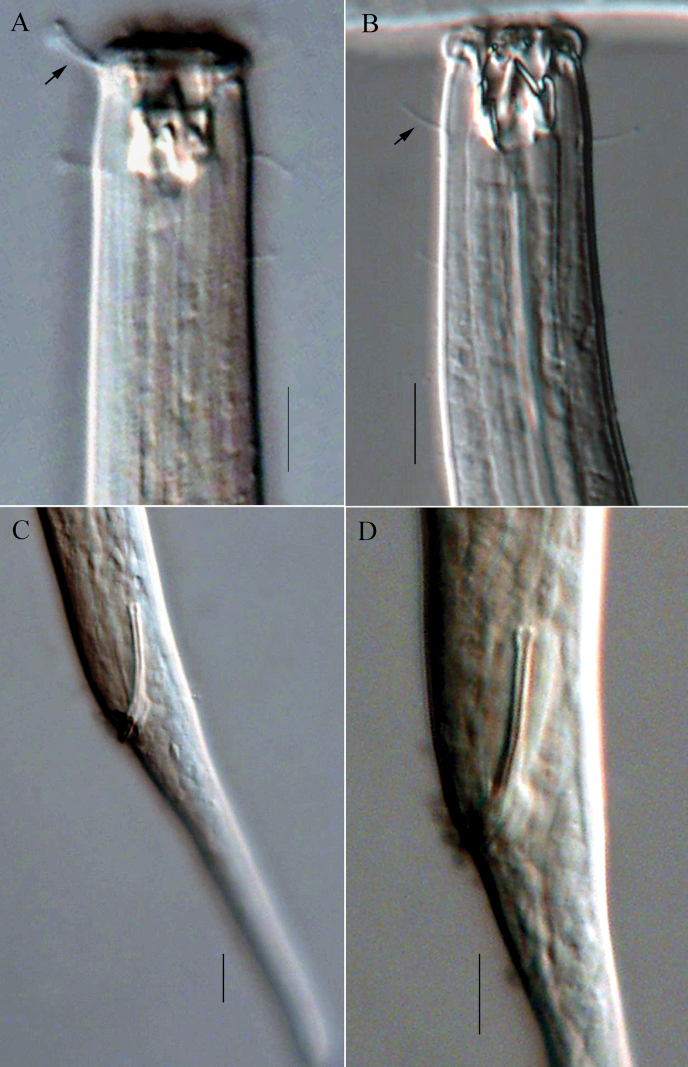
Micrographs of *Viscosiamedia* sp. nov. **A** anterior end of holotype, showing outer labial and cephalic setae (arrow); buccal cavity with three teeth **B** anterior end of female, showing right subventral tooth and cervical setae (arrow) **C** posterior end of holotype, showing spicule and tail **D** cloacal region of male, showing spicule. Scale bars: 10 µm.

**Figure 3.**
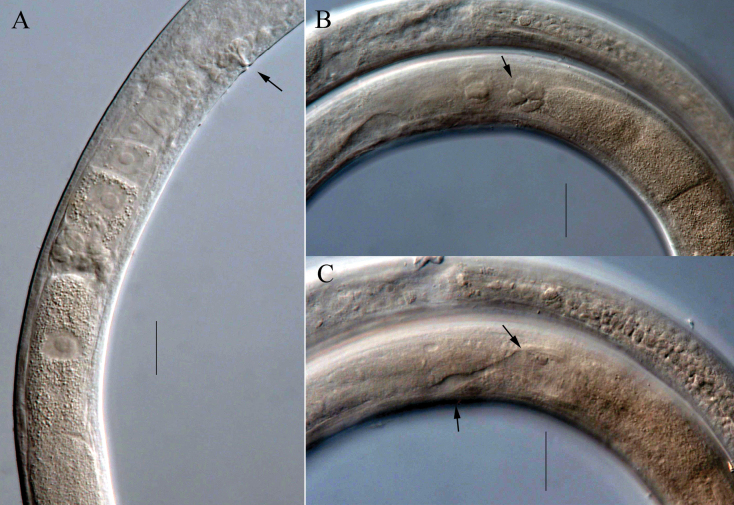
Micrographs of *Viscosiamedia* sp. nov. **A** middle region of female body, showing vulva (arrow), ovary and eggs **B** showing gland cells (arrow) at the reflexed point of ovary **C** showing sac-like structure and osmosium of Demanian system (arrows).

Figs 1 , 2 , 3 , Table 1

##### Diagnosis.

Body slender, medium size in the genus. Heavily cuticularized and relatively shallow buccal cavity with three stubby teeth, and a right ventrosublateral tooth massive. Cephalic setae 7–8 µm long. Amphidial fovea invisible. Spicules slender, almost straight, cephalated proximally and conical distally. Tail conical, straight in males, slightly bent ventrally in females.

##### Holotype and paratype material.

Four males and two females were measured. ***Holotype*** male 1 on slide RZ080312-9. ***Paratype*** 1 (male 2) on slide RZ080310-3, both ***paratype*** 2 (male 3) and ***paratype*** 3 (male 4) on slide RZ0803123-4, ***paratype*** 4 (female 1) on slide RZ080312-9, and ***paratype*** 5 (female 2) on slide RZ0803123-4.

##### Type locality and habitat.

Holotype and paratypes were all collected from the surface layer of fine sand sediment on an intertidal beach along the Rizhao coast of the Yellow Sea, China (35°34'21"N, 119°39'29"E).

##### Etymology.

Species epithet *media* refers to the medium body size of this species within the genus.

##### Measurements.

All measurement data are given in Table 1.

**Table 1.** Individual measurements of *Viscosiamedia* sp. nov. and *Viscosiasinica* sp. nov. (in µm except a, b, c, c′ and V%) a, ratio of body length to maximum body diameter; b, ratio of body length to pharynx length; c, ratio of body length to tail length; c′, ratio of tail length to cloacal or anus body diameter; V%, position of vulva from anterior end expressed as a percentage of total body length; -, no data.

Characters	*Viscosiamedia* sp. nov.						*Viscosiasinica* sp. nov.					
	♂1	♂2	♂3	♂4	♀1	♀2	♂1	♂2	♂3	♂4	♀1	♀2
Total body length	1620	1534	1615	1522	1539	1458	1733	1885	1840	1821	1457	1653
Maximum body diameter	32	33	33	26	41	35	28	26	26	25	31	28
Head diameter	17	16	17	16	18	17	13	14	14	14	13	15
Length of outer labial setae	6	7	7	6	6	6	5	7	7	5	5	6
Length of cephalic setae	7	8	7	7	7	6	5	7	7	5	5	6
Depth of buccal cavity	18	15	16	17	16	17	29	22	21	26	16	16
Width of buccal cavity	9	8	8	7	9	9	10	8	8	8	8	9
Height of amphidial fovea	-	-	-	-	-	-	5	5	6	5	4	5
Width of amphidial fovea	-	-	-	-	-	-	10	11	11	9	8	8
Length of pharynx	317	310	320	307	305	312	278	298	287	304	260	304
Body diameter at pharyngeal base	26	27	27	24	32	31	24	23	24	24	31	26
Spicules length along arc	29	24	30	32	-	-	20	21	22	19	-	-
Vulva from anterior end	-	-	-	-	732	690	-	-	-	-	720	820
V%	-	-	-	-	47.6	47.3	-	-	-	-	49.4	49.6
Body diameter at cloaca or anus	17	16	17	15	16	15	16	16	16	16	19	15
Tail length	86	75	85	80	75	83	81	104	106	98	88	97
a	50.6	46.5	48.9	58.5	37.5	41.7	61.9	72.5	70.8	72.8	47.0	59.0
b	5.1	4.9	5.1	5.0	5.0	4.7	6.2	6.3	6.4	6.1	5.6	5.4
c	18.8	20.4	19.0	19.0	20.5	17.6	21.4	18.1	17.4	18.6	16.6	17.0
c′	5.1	4.7	5.0	5.3	4.7	5.5	5.1	6.5	6.6	6.1	4.4	6.5

##### Descriptions.

**Males.** Body medium in size, and slender, tapering slightly towards both extremities. Cuticle smooth. Cervical setae 4–6 µm long, sparse, present only in the anterior portion of pharyngeal region. Cephalic region truncated, continuing with body contour. Six lips, each bearing a single inner labial papilla. Six outer labial setae and four slightly longer cephalic setae in a single circle. Outer labial setae 6–7 µm long, cephalic setae 7–8 µm long. Amphidial fovea invisible. Buccal cavity heavily cuticularized, relatively shallow, 15–18 µm deep and 7–9 µm wide, with three stubby teeth. Right ventrosublateral tooth massive and larger than left ventrosublateral tooth and dorsal tooth. The tip of right ventrosublateral tooth at the same level with outer labial and cephalic setae. The height of left ventrosublateral tooth and dorsal tooth are almost equal. Pharynx cylindrical, widening slightly towards posterior extremity. Cardia conical, surrounded by intestinal tissue. Nerve ring located pre-mid of length of pharynx. Secretory-excretory system present; excretory pore located slightly posterior to nerve ring, 160–170 µm from anterior end. Ventral gland located at anterior part of the intestine.

Reproductive system with two opposed and outstretched testes located to the right of intestine. Sperm cells oval or irregularly square, 12–16 µm long and 8–10 µm wide. Spicules slender, almost straight, cephalated proximally and conical distally. Gubernaculum absent. Tail conical, narrowing abruptly immediately posterior to cloaca and directed slightly dorsally, without caudal setae. Three caudal glands extending anteriorly to tail region. Spinneret present.

**Females.** Similar to males in most morphological characteristics but there are less cervical setae, and tail slightly bent ventrally. Reproductive system with two opposed and reflexed ovaries both located to the right of intestine. Eggs very long, can be up to 220 µm long. Demanian system simple, consisting of a sac-like prolongation of the ovary at the reflexed point of the ovary and a duct-shaped osmosium. Vulva located at mid-body.

##### Differential diagnosis and discussion.

*Viscosiamedia* sp. nov. is characterized by heavily cuticularized and relatively shallow buccal cavity with three stubby teeth, right ventrosublateral tooth massive, cephalic setae 7–8 µm long, amphidial fovea invisible, spicules slender, almost straight, cephalated proximally and conical distally, tail conical, straight in males, slightly bent ventrally in females. The new species is similar to *V.epapillosa* Platonova, 1971 in the deep of buccal cavity and length of cephalic setae, but differs by the wider head diameter (16–18 µm *versus* 11–13 µm), buccal cavity with heavily cuticularized parallel walls (*versus* buccal cavity wider in the middle and narrower at the ends), having cervical setae (*versus* absent in the latter species), spicules enlarged proximally (*versus* not enlarged), and a conical tail almost straight in the males (*versus* conico-cylindrical tail bent in the latter species). The new species is also similar to *Viscosiapygmaea* Nguyen Vu Thanh & Gagarin, 2013 in the tail shape, but differs from *V.pygmaea* by the longer body length (1458–1620 µm *versus* 838–992 µm), longer cephalic setae (7–8 µm *versus* 3–3.5 µm), shallower buccal cavity with stubby teeth (1 head diameter *versus* 1.5 head diameters in depth with slender teeth), amphidial fovea invisible (*versus* obvious and located close to the base of buccal cavity), and the different spicule shape (slender with cephalated proximal end and tapering distally end *versus* conical and tapering off). *Viscosiamedia* sp. nov. can be differentiated from all other species of the genus by its heavily cuticularized and relatively shallow buccal cavity with stubby teeth, 7–8 µm cephalic setae, and a conical tail. The basic morphological characteristics of the valid species with similar size to the new species in the genus are compared in Table 2.

**Table 2.** Basic morphological features of males of the valid species in *Viscosia* with body length of 1000–2900 µm (according to Gagarin 2020) (in µm except a, b, c and c′) L, body length; a, ratio of body length to maximum body diameter; b, ratio of body length to pharynx length; c, ratio of body length to tail length; c′, ratio of tail length to cloacal or anus body diameter.

Species	L	a	b	c	c′	Cephalic sensilla length	Buccal cavity depth	Spicules length
*V.bandaensis*	1389–1715	41–48	4.8–5.1	12.8–13.8	6.0–6.5	papilla	23	18–20
*V.bayensis*	1630–1820	41–48	5.2	17.3	5.6	9–11	25–29	43–50
*V.dossena*	1759–2048	23–25	5–6	19–20	3.0	4–6	31–35	27–33
*V.epapillosa*	1750	51	5.3	20.7	4.9	6	16	29
*V.erasmi*	1905	60	5.7	15.9	5.0	5	23	32
*V.glabra*	1600–2100	42–70	5.3–6.3	7.5–11.0	10–13.0	papilla	23–25	27
*V.longicaudatoides*	1348–1432	53–54	4.8–5.4	8.3–9.0	10.1–10.8	2.3–3	17–18	27
*V.macramphida*	1400	39	5.6	7.9	9.6	papilla	15	18–19
*V.macrobursata*	1680–2200	50–53	5.7	13.2	8.6	10–12	23–29	20
*V.meridionalis*	1415–1743	30–43	5.3–6.9	8.2–9.6	7.3–9.8	papilla	18–25	32–40
*V.media* sp. nov.	1522–1620	46.5–58.5	4.9–5.1	18.8–20.4	4.7–5.5	7–8	16–18	24–30
*V.microseta*	1850	44	6.1	16.5	4.3	1.5–2.0 µm	20–22	33
*V.nuda*	1625–1931	45–54	5.2–6.0	7.3–8.0	13.0–13.5	papilla	18–22	18
*V.oncholaimelloides*	1950	89	6.5	13.9	9.1	papilla	13	17
*V.orientalis*	991–1122	17–20	4.7–5.1	15.7–19.9	2.8–3.0	2.0 µm	18–20	34–36
*V.papillatoides*	1520–2200	40–51	5.5–5.6	19.1–23.0	3.4–4.6	papilla	24–32	26–29
*V.papillata*	1520	31	5.0	11.0	6.0	papilla	18	24
*V.parasetosa*	1721–1748	52–55	5.7–5.9	11.6–12.0	8.2–8.3	2.7 µm	18–20	24
*V.profunda*	1311–1501	47–50	5.0–5.6	14.8–15.4	4.5–5.2	2.0–2.5 µm	17.5–18	17.5
*V.separabilis*	1320–2200	32–78	4.8–7.8	12.9–16.6	5.0–8.0	7.5 µm	20–26	19–26
*V.setosus*	1784–1857	51–57	6.0–6.1	11.9–12.1	8.0–9.0	5.0 µm	20–21	22
*V.sinica* sp. nov.	1733–1885	62–73	6.1–6.4	17.4–21.4	5.1–6.6	5–7 µm	26–29	19–22
*V.stenolaimus*	1870	37	4.5	12.6	4.6	papilla	25–27	37
*V.timmi*	1016–1509	22–37	4.1–5.1	16.0–25.6	2.0–3.5	2.5–3.0 µm	21–28	24–28
*V.viscosa*	1700–2600	52–60	5.0–6.5	12.0–16.0	6.0–7.5	4.0 µm	22–30	27–30
*V.weiseri*	1600–2900	29–46	4.3–5.2	13.3–17.7	4.5	5.0 µm	28–30	42

#### ﻿ Viscosia sinica sp. nov.

Taxon classificationAnimaliaEnoplidaOncholaimidae

53BDE026-D625-52D3-B83A-E1176C7DF176

https://zoobank.org/1EB9C0EF-BDC5-484C-8932-4CFBA8F1D88D

**Figure 4.**
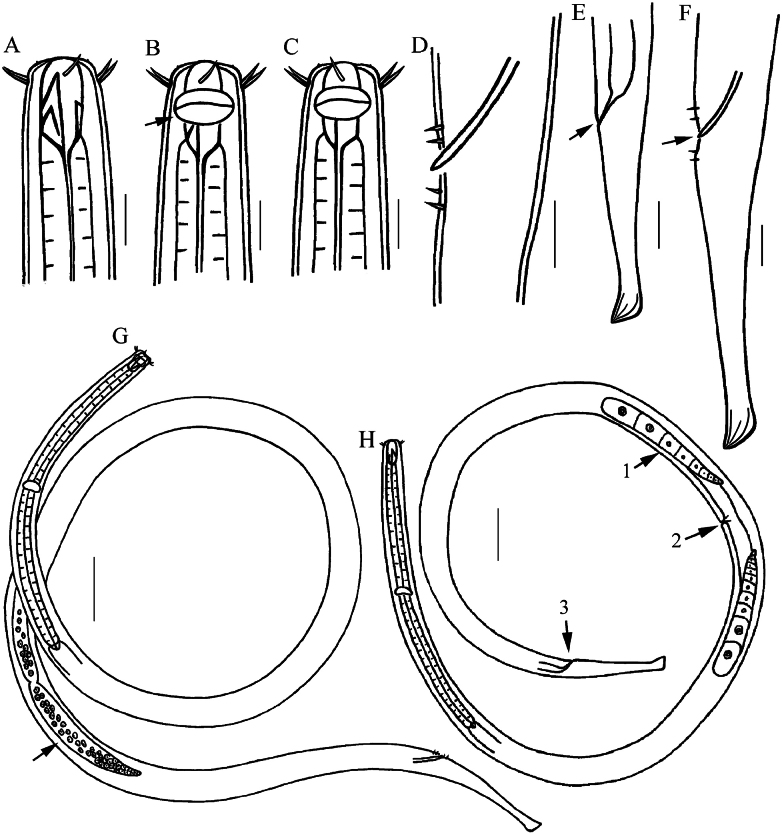
Drawings of *Viscosiasinica* sp. nov. **A** anterior end of holotype, showing outer labial and cephalic setae, buccal cavity with three teeth **B** anterior end of male, showing amphidial fovea (arrow) **C** anterior end of female **D** spicule and setae surrounding the cloaca **E** posterior end of female, showing anus (arrow) and tail **F** posterior end of holotype, showing cloaca (arrow), spicule and tail **G** entire view of male (arrow showing testis) **H** entire view of female (arrow 1, ovary; 2, vulva; 3, anus). Scale bars: 10 µm (**A–D, F**); 20 µm (**E**); 50 µm (**G, H**).

**Figure 5.**
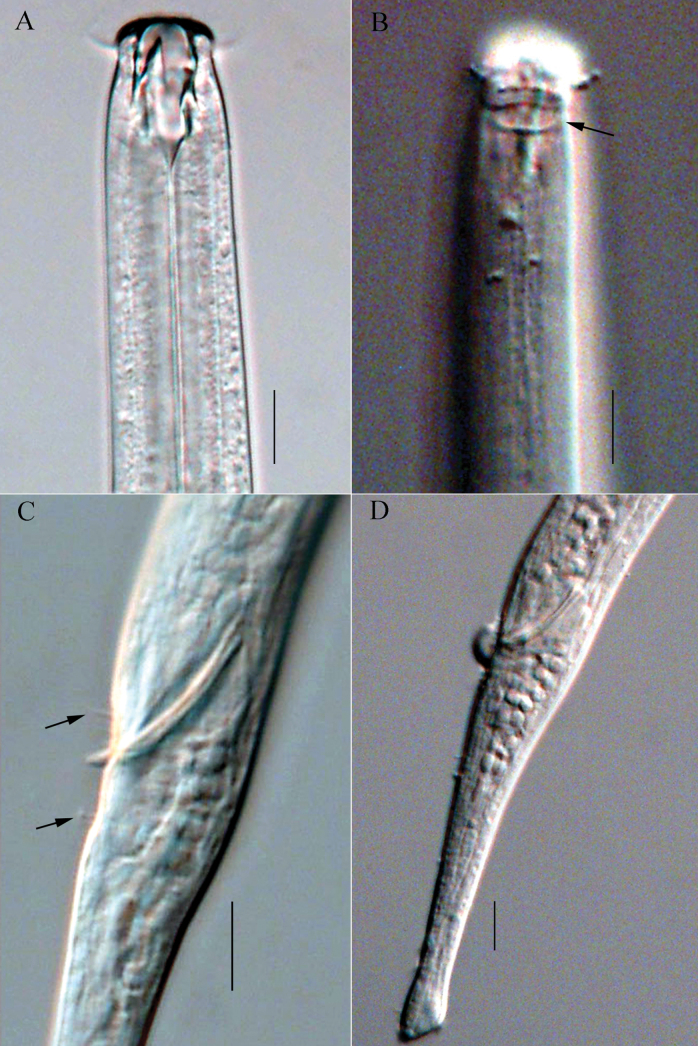
Micrographs of *Viscosiasinica* sp. nov. **A** anterior end of holotype, showing buccal cavity with large right subventral and dorsal teeth **B** anterior end of male 1, showing outer labial and cephalic setae, amphidial fovea (arrow) **C** cloacal region of male 2, showing spicule and setae surrounding cloaca (arrows) **D** posterior end of holotype, showing spicule and tail. Scale bars: 10 µm.

Figs 4 , 5 , Table 1

##### Dianosis.

Relatively large amphidial fovea, about 80% corresponding body diameter wider in males and 53–62% in females. Cephalic setae 5–7 µm long. Tail conico-cylindrcal with swollen horseshoe-shaped tip. Spicules slender, slightly curved ventrally, not cephalate proximally. 10–12 setae surrounding the cloaca, each 3–4 µm long.

##### Holotype and paratype material.

Four males and two females were measured. ***Holotype*** male 1 on slide RZ080123-4. ***Paratype*** 1 (male 2) on slide YST24251-2, paratype 2 (male 3) on slide YST24381-11, paratype 3 (male 4) on slide YST24253-1, paratype 4 (female 1) on slide RZ0803123-4, paratype 5 (female 2) on slide YST2418-12.

##### Type locality and habitat.

Holotype and paratype 4 (female 1) were collected from the surface layer of fine sand sediment on an intertidal beach along the Rizhao coast of the Yellow Sea (35°34'21"N, 119°39'29"E). The other paratypes were all collected from the surface layer of silt sediment on an intertidal beach of Huangdao along the Yellow Sea (31°44'53"N, 121°55'43"E).

##### Etymology.

The species epithet refers to the country of origin, China.

##### Measurements.

All measurement data are given in Table 1.

##### Descriptions.

**Males.** Body relatively slender. Cuticle smooth, without somatic setae. Labial region spherical, demarcated by slight constriction. Six lips, each bearing a single internal labial papilla. Six outer labial setae and four cephalic setae in a single circle, equal in length. Amphidial fovea relatively large, pocket-shaped, 9–11 µm wide, about 80% corresponding body diameter, and 5–6 µm deep, located at level of the middle of buccal cavity. Buccal cavity 16–29 µm deep and 8–10 µm wide, with three teeth. Right ventrosublateral tooth larger than left ventrosublateral tooth and dorsal tooth. The tip of right ventrosublateral tooth at the same level with outer labial and cephalic setae. The height of left ventrosublateral tooth and dorsal tooth are almost equal. Pharynx cylindrical, widening slightly towards posterior extremity. Cardia conical, about 15 µm long, surrounded by intestinal tissue. Nerve ring located approximately halfway down length of pharynx. Secretory-excretory system and excretory pore not observed.

Reproductive system with two opposed and outstretched testes located to the right of intestine. Spicules slender, slightly curved ventrally, not cephalate proximally. Gubernaculum absent. 10–12 setae surrounding the cloaca, each 3–4 µm long. Tail conico-cylindrical, with swollen horseshoe-shaped tip. Caudal setae absent. Three caudal glands extending anteriorly to tail region. Spinneret present.

**Females.** Similar to males in the most morphological characteristics but amphidial fovea slightly smaller, 53–62% corresponding body diameter wider, and buccal cavity slightly shallower (21–29 µm *versus* 16 µm deep). Reproductive system with two opposed and reflexed ovaries both located to the right of intestine. Demanian system indistinct. Vulva located at mid-body.

##### Differential diagnosis and discussion.

*Viscosiasinica* sp. nov. is characterized by relatively large amphidial fovea, conico-cylindrcal tail with horseshoe-shaped tip, spicules slender, slightly curved ventrally, not cephalate proximally, 10–12 setae surrounding the cloaca, each 3–4 µm long. The new species resembles *V.brachylaima* Filipjev, 1927 and *V.filipjevi* Paramonov, 1929 in tail shape, but differs from *V.brachylaima* by larger amphidial fovea (80% *versus* 30% corresponding body diameter) and higher value of de Man ratio *a* in males (61.9–72.8 *versus* 42.5). The new species differs from *V.filipjevi* which description is based only in females by larger amphidial fovea (wider than width of buccal cavity *versus* narrower than width of buccal cavity), and smaller body size (1.46–1.65 mm in body length and 28–31 µm maximum body diameter in females *versus* 2.17–2.20 mm in length and 40.5–48.6 µm maximum body diameter in *V.filipjevi*). The new species is also similar to *V.elegans* (Kreis, 1924) Lorenzen, 1981 in body size and shape, but differs by larger amphidial fovea (80% *versus* 33% corresponding body diameter) and horseshoe-shaped tail tip. The new species differs from *V.media* sp. nov. in having deeper buccal cavity with slender teeth in males (21–29 µm deep *versus* 15–18 µm with stubby teeth), larger amphidial fovea (*versus* invisible), and different tail shape (conico-cylindrical with swollen horseshoe-shaped end *versus* conical). *Viscosiasinica* sp. nov. is distinguished from all other known species of the genus by its relatively large amphidial fovea, 5–7 µm cephalic setae, and conico-cylindrical tail with swollen horseshoe-shaped end.

## Supplementary Material

XML Treatment for Viscosia

XML Treatment for Viscosia media

XML Treatment for Viscosia sinica

## ﻿Additional information

### Author ORCIDs

Lingyun Sun https://orcid.org/0009-0005-3553-3645

Huimin Gu https://orcid.org/0009-0008-5820-4678

Yong Huang https://orcid.org/0000-0002-1846-8088

### Data availability

All of the data that support the findings of this study are available in the main text.
